# Role of Genetics in Early-Onset Cardiovascular Disease

**DOI:** 10.7759/cureus.85988

**Published:** 2025-06-14

**Authors:** Muhammad Meer, Muhsina Meer, Mahnoor Mumtaz, Umar Jawed

**Affiliations:** 1 Acute Internal Medicine, Northampton General Hospital, Northampton, GBR; 2 Family Medicine, Shabbir Suleman Meer and Partners, Pinetown, ZAF; 3 Emergency Medicine, Northampton General Hospital, Northampton, GBR; 4 Internal Medicine, Northampton General Hospital, Northampton, GBR

**Keywords:** early-onset cardiovascular disease, genetic predisposition, genetic testing and personalized medicine, monogenic disorders, polygenic risk score

## Abstract

Genetics increasingly comes to the front with early-onset cardiovascular disease (CVD) since researchers investigate the complex interplay of hereditary factors that promote an early manifestation of the disease. CVD is one of the most general causes of morbidity and mortality worldwide, presenting unique challenges when it arises in younger populations many times due to genetic predispositions. The various etiologies in the pathogenesis of early-onset CVD involve genetic factors, including the monogenic disorders of familial hypercholesterolemia (FH) and hypertrophic cardiomyopathy (HCM) of these diseases showing the simple Mendelian patterns of inheritance. These may be mediated through gene variations, including Low-Density Lipoprotein Receptor (LDLR), Apolipoprotein B (APOB), Proprotein Convertase Subtilisin/Kexin Type 9 (PCSK9), and Myosin Heavy Chain 7 (MYH7). Disrupted lipid metabolism, myocardial function, or vascular integrity due to mutations could lead to adverse clinical consequences. Moreover, polygenic risk score (PRS) has now become helpful in identifying individuals who are at elevated risk due to the cumulative effect of several genetic variants. Knowledge about gene-environment interactions, epigenetic influences, and complex regulatory networks contributes to understanding the importance of genetic contributions to early-onset CVD. However, the genetic variation is population-specific and underlines the need for research inclusive of diverse genetic backgrounds in developing more inclusive and effective predictive models. Whole genome and exome sequencing have revolutionized early detection, making personalized treatment plans possible, including targeted therapeutic interventions like PCSK9 inhibitors. On the other hand, such scientific progress also provides a lot of ethical challenges, such as utilizing personal data, informed consent, and equal access to genetic services. This review summarizes the genetic basis underlying early-onset CVD, with detailed discussions of monogenic and polygenic contributions, important genetic pathways, and emerging advances in genetic testing and personalized medicine approaches. By highlighting the integration of genetic insights with preventive and therapeutic strategies, this review aims to bring into focus the use of genetic insight in the betterment of outcomes in patients and inform future research in cardiovascular genetics.

## Introduction and background

Cardiovascular disease (CVD) is the major cause of sickness and death worldwide, ranking at the top in terms of burden [1]. In the US completely, about 62 million people are diagnosed with a certain form of CVD, whereas 50 million people are living with hypertension [2]. Known from epidemiological studies, CVD contributed to 946,000 deaths in 2000, accounting for 39% of all deaths [3]. These alarming statistics point to the pervasive nature of the cardiovascular condition and heighten the need for comprehensive prevention and intervention strategies [4].

Whereas the well-acknowledged risk factors for developing CVD include lifestyle aspects related to diet, physical inactivity, and smoking, the underlying genetic propensity forms an equally important yet complex contribution to its incidence-even in younger cohorts [5]. A heritable component of CVD increasingly has been recognized as central to understanding the pathophysiology and progression of various cardiovascular conditions [6]. Although rare, monogenic forms (caused by mutations in a single gene, e.g., familial hypercholesterolemia (FH)) of cardiovascular disorders have provided an important framework for identifying the various molecular pathways and mechanisms underlying more common and complex forms of disease [7].

The early studies of FH and other single-gene disorders have provided insight into the ways that a mutation in a single gene can lead to extreme elevations in low-density lipoprotein (LDL) cholesterol and premature coronary artery disease (CAD) [8]. Further, the inherited forms of hypertension and cardiac arrhythmias have unraveled the genetic mutation of ion channels and transporters, underlining their critical role in disease pathogenesis [9]. These findings depict that, while most cardiovascular disorders result from complex interactions of genes and the environment, assessments of less common genetic variants provide critical information on clinical diagnosis and selective therapies [10]. This review will discuss genetic susceptibility to early-onset CVD emphasizing the lessons learned from the genetics of monogenic disorders and gene variants associated with complex traits, implicating molecular diagnosis and patient management [11]. It will extensively explain how hereditary factors influence early cardiovascular conditions and how they might be used to benefit syntax patients in terms of better outcomes and prevention strategies [5].

While this review prioritizes disorders with predominant early-onset manifestations (e.g., FH, hypertrophic cardiomyopathy (HCM)), we also discuss conditions with variable age of presentation (e.g., Marfan syndrome, Lamin A and C (LMNA) cardiomyopathies) when they exhibit life-threatening cardiovascular complications in young populations. These inclusions are justified by their mechanistic insights into shared pathways (e.g., aortic fragility, arrhythmias) and clinical urgency in high-risk youth, even if penetrance is incomplete.

Significance of early-onset CVD

Early-onset CVD is of great concern because of its huge burden on global health, as most CVD-related deaths occur in low- and middle-income countries [12]. Classically regarded as a disease in developed regions such as the US and western Europe, CVD rates have recently begun to decline there due to improved prevention and treatment strategies [13]. Contrary to that, CVD cases are increasing very rapidly in developing countries due to a rise in age-specific mortality rates resulting from changes in lifestyle, less access to health facilities, and fewer early detection systems [14].

Early-onset CVD is characterized by the presence of cardiovascular conditions at an early age and thus has particular challenges. It contributes not only to high morbidity and mortality but also shares a significant economic burden on healthcare systems that are already resource-constrained [15]. Risk factors such as hypertension, smoking, poor diet, and obesity have a more pronounced influence on the rise of CVD as countries undergo urbanization and economic growth; this characterizes the shift in prevailing diseases from predominantly infectious diseases to non-communicable diseases such as CVD [16].

The risk factors for CVD need to be identified early for their effective management or intervention. Unfortunately, despite recent advances in various diagnostic imaging modalities and screening methodologies, these are seldom utilized for regular checks in developing countries as a result of budgetary constraints, insufficient trained personnel, and poor health infrastructure [16]. The capability to identify functional and structural alterations in the myocardium and vasculature, even before the onset of symptoms, may lead to dramatic improvement in outcomes, thus facilitating early and cost-effective interventions [17]. Further, prevention strategies involving lifestyle changes and prophylactic treatments have shown a reduction in the progression toward serious disease, which just goes to reiterate how important early modification of CVD can be for reducing its long-term burden [1].

The complicated nature of nonlinear interactions and dynamic regulatory networks in the genetic components of CVD makes the factors very difficult to understand [6]. These interactions do not take place in isolation; epigenetic influences and a lifetime of environmental exposures mold the way they function [18]. These multidimensional interrelationships between genotype and phenotype underscore the need for comprehensive genetic "profiling" as part of the research into the etiology of diseases in the study of CVD. Such an approach shall go a long way in improving predictive models [19].

Moreover, the genetic variations responsible for CVD are population-specific due to genetic background and environmental exposure peculiar to different populations [20]. Depending on this fact, this population specificity further narrows the possibility of generalization across populations through the use of tailored research strategies that take into consideration diverse genetic and environmental contexts [21]. Lack of recognition and factors in such complexity may result in simplistic predictive modeling and therapeutic strategy that fails to take into account important interactions driving early-onset CVD [22].

Any consideration of the genetic factors in CVD cannot be limited to reductionist models that isolate single causal agents but must adopt a more holistic approach that considers a range of genes and environmental agents interacting in complex networks over time [23]. Overcoming all these challenges will help to advance this area of research into CVD by identifying persons at high risk while informing specific targeted interventions in the effort to prevent the early development and progression of the disease [16].

Background

Early onset CVD is defined as cardiovascular conditions manifesting at an earlier age compared with the conventional outlook, mostly with devastating and sudden results [11]. For example, HCM is considered to be the most common monogenic cardiac disorder and a leading cause of sudden death in children and adolescents. Its incidence is around 1 in 500 individuals [24]. Such cases depict a range of genetic predispositions that are linked with early onset CVD [7].

Monogenic diseases due to a mutation in one gene are important models for genetic studies of early cardiovascular manifestations [25]. Indeed, these are usually caused by mutations in genes that functionally codify critical cardiac functions, including myocardial contractility and ion channel activity [9]. For example, mutations of genes encoding sarcomeric proteins may lead to HCM, while abnormalities in some other genes such as Sodium Voltage-Gated Channel Alpha Subunit 5 (SCN5A) or KVLQT1 may cause arrhythmias, which may turn out to be life-threatening in early life [26,27]. Accordingly, while monogenic disorders provide a clear link between genetics and early onset CVD, research has shown that complex genetic and environmental interactions also underpin such early presentations [6]. Knowledge of such genetic underpinnings will not only inform clinical diagnosis but also support the development of preventive and therapeutic measures tailored to individuals at high risk of early-onset CVD [5].

The incidence and prevalence of early-onset CVD have shown disturbing trends over the most recent decades and remain particularly evident in developed areas [4]. The rise in early-onset CAD reflects gene-environment interactions, where genetic risks (e.g., Low-Density Lipoprotein Receptor (LDLR) mutations) are exacerbated by modifiable factors like obesity, poor diet, and physical inactivity [13]. In contrast, cardiomyopathies like HCM arise from sarcomere mutations (e.g., Myosin Heavy Chain 7 (MYH7)) independent of lifestyle factors [24]. Data illustrate that, in contrast to the declining trends for CVD among older adults, the incidence and prevalence of various forms of early-onset CVD among young adults have remained steady or increased over the last few decades [12].

For instance, a report by the Worcester Heart Attack Study indicated that the rates of myocardial infarction in residents less than 55 years old remained stable between the mid-1980s and 2005 [28]. A cohort study in Denmark also estimated that the incidence of heart failure in such patients aged less than 50 years increased, where there was a 50% reduction in incidence in people of the older population during the same time interval [29]. In France, the hospitalization rates of women under the age of 65 for myocardial infarction increased by 6% between the years 2004 and 2014, whereas no change was noted in men of that age bracket [30]. The Atherosclerosis Risk in Communities (ARIC) study revealed ethnicity-related differences in the incidence of myocardial infarction among participants aged 35-44 years: Black men, Black women, White men, and White women [3]. In Australia, the rates of myocardial infarction among participants below the age of 50 years did not change between 1993 and 2012 [31].

The analysis of temporal trends in CVD among young adults shows a mixed picture by age group. For example, while the prevalence of CAD fell from 1.6 to 1.2% for those aged 18-44 years in the US between 2006 and 2010, other studies show that these declines are not as sharp or consistent across the world [32]. For example, in countries like Denmark and Norway, CVD rates among those aged less than 50 years have remained relatively stable or have indeed slightly increased, reflecting a trend of non-declining rates contrasting with the usual declining rates seen in their elderly populations [33].

Early-onset CVD: risk factors and genes

Early onset of CVD among young adults is attributed to both modifiable and non-modifiable risk factors [34]. Traditional cardiovascular risk factors, including smoking, obesity, hypertension, and diabetes, have a high burden among the young [35]. For example, the rates of obesity have tripled in less than 40 years among the youthful population and play an important role in contributing to CVD risk [36]. Moreover, a sedentary lifestyle, one of the leading factors of cardiovascular risk, affects more than 30% of young adults in the US, which is similarly observed in other global regions as well [37].

Other unhealthy developments in substance abuse, including opioid use and anabolic steroids, now also contribute to increased CVD risk among young adults [38]. Centres for Disease Control and Prevention (CDC) reports mentioned that heroin use and opioid-related deaths have more than doubled in the last decade among those aged 18-25 years [39]. The prevalence of smoking, while continuing to fall globally, remains high in younger age groups and is thereby contributing to future CVD risk [40].

Genetic contributions to CVDs were first recognized in the form of inherited patterns seen in selected cardiac conditions, of which HCM and FH are two notable disorders [40]. The notion of monogenic disorders, such as FH, in which more than 600 mutations in the LDLR gene were identified, provided early insights into the molecular mechanism for CVD [41]. Single-gene mutations including these genes were followed by research into the ways LDLR deficits could result in a drastic increase in plasma cholesterol levels and an early onset of CAD [42]. 

Similarly, mutations in genes coding for Apolipoprotein B-100 (APOB-100) and ATP-binding cassette transporters (ABCG5 and ABCG8) underlined genetic pathways influencing cholesterol absorption and clearance, toward which targeted therapies, including the use of statin treatment, molded their foundation [43]. Genetic studies eventually moved from solely investigating these simple Mendelian cardiovascular disorders to those involving more complex traits [44]. Genetic studies have identified mutations in genes encoding myocardial contractile proteins such as β-myosin heavy chain and cardiac myosin-binding protein C, which facilitate further understanding of HCM and its variable clinical manifestations [45].

Research about the causes of inherited arrhythmias underlined again the important role of genetic variants in cardiac ion channels. For example, variants in the SCN5A gene were implicated in conditions such as long-QT syndrome and ventricular fibrillation [46]. These genetic studies gave valuable models for disease mechanisms and resulted in more discriminating approaches to clinical diagnosis and risk stratification. Overall, these aforementioned studies have significantly advanced the field of cardiovascular genetics [47].

## Review

Methodology

Literature Search Strategy 

A literature search was performed to comprehensively study the role of genetics in early-onset CVD. The primary databases utilized included PubMed, Google Scholar, and Scopus, considering their extensive repository of biomedical and clinical research articles. The search strategy was targeted toward the retrieval of peer-reviewed articles in the English language published up to 2023. The following keywords and phrases were considered, individually and in combination, to narrow the results: "early onset cardiovascular disease," "genetic predisposition to CVD," "monogenic cardiovascular disorders," "genetic mutations in heart disease," "familial hypercholesterolemia," "hypertrophic cardiomyopathy genetics," and "arrhythmia genetic factors.".

Other specific review inclusion criteria included articles that addressed the review question in terms of the genetic basis of CVD in young adults or children. Also, studies discussing the prevalence and impact of specific gene mutations or polymorphisms associated with early-onset CVD were considered, as well as research into genetic screening and risk assessment. The exclusion criteria included articles that were based solely on environmental or lifestyle factors, studies in which there was inadequate involvement of genetics, or those completely related to the older population without any early-onset CVD.

Review Methodology 

Articles and data were analyzed critically in several stages. The abstracts were read to identify relevance for the review based on the criteria for inclusion and exclusion outlined. Full-text articles were then read to identify the key findings related to genetic mutations, molecular pathways, and their clinical implications in early-onset CVD. Particular emphasis was given to those studies that reported genetic linkage analyses, Genome-Wide Association Study (GWAS), and those studies that depicted monogenic and polygenic contributions to CVD.

Each identified study was analyzed for the specific cardiovascular condition being discussed. Synthesis of the findings through data extraction is done to provide an overview of current knowledge and research gaps and areas still in need of study. This is a comprehensive approach that covers foundational studies, as well as the most recent developments in cardiovascular genetics.

Genetic basis of early-onset CVD

Genetic background can be related to CVD in many ways. Investigations have emphasized that most early-onset CVDs can be attributed to monogenic disorders, largely associated with the mutations of a single gene. FH is one such well-recognized monogenic disorder caused by a mutation in LDLR, APOB, and Proprotein Convertase Subtilisin/Kexin Type 9 (PCSK9) genes responsible for inducing high levels of LDL cholestrol and early atherosclerosis [7]. Because these mutations are fully penetrant, they are important for the identification of individuals at risk for premature atherosclerotic disease [7,48].

In contrast, the majority of early-onset CVD cases result from polygenic inheritance, where multiple genetic variants each contribute rather modestly to the overall risk [5]. Because of this, polygenic risk scores (PRSs) are calculated from genome-wide association studies to integrate the cumulative effect of these variants. To date, the application of PRS has been useful in identifying individuals who are at higher risk and who do not carry a single high-penetrance mutation but who have significant risk due to their aggregate genetic profile [49,50].

Monogenic diseases like FH are representative of the profound, direct contribution that known gene variants have on CVD risk. Generally, subjects with FH display very high levels of cholesterol very early in life and a greatly enhanced risk of myocardial infarction at relatively young ages in the absence of treatment [7]. A study shows that mutations for FH alter cholesterol metabolism and support a role for early genetic testing in families at risk [48].

The contribution of polygenic risk cannot be underestimated in early-onset CVD. PRS marries the effects of many variants to yield an estimate of the genetic risk that an individual carries to CVD. These scores extend risk stratification beyond traditional risk factors, enabling preventive strategies even in individuals who do not have overt monogenic mutations [5,51]. Recent reports emphasize the view that individuals falling in the top percentile of PRS distributions are at risks comparable to, or exceeding, those with monogenic conditions like FH [52]. 

Clinical practice with PRS will eventually highlight those at high risk for early interventions that might modify the course of a disease or condition [11]. However, there are challenges in establishing the general applicability of PRS across diverse populations and in refining its integration into personalized healthcare [6,53].

Key genes and pathways in early-onset CVD

The genetic factor, especially the mutations and variants in the LDLR, APOB, and PCSK9 genes, is very important in the development of early-onset CVD. These genes are crucial in the regulation of lipid metabolism, and their disturbing action leads to increased susceptibility to developing CVD by modulating cholesterol levels and other lipoprotein metrics.

Low-Density Lipoprotein Receptor

The LDLR gene encodes a crucial constituent, the low-density lipoprotein receptor. This constituent plays a major role in mediating the endocytosis and clearance of LDL cholesterol from the circulation. Mutations within this gene constitute a common cause of FH, wherein subjects maintain high levels of LDL cholesterol and an increased susceptibility to the premature development of atherosclerosis. [54]. Variants in LDLR typically result in the impaired function of the receptor or reduced expression, leading to a reduced clearance of LDL from the circulation. Conclusively, it may lead to an accumulation of lipids and the development of plaques in arterial walls [55].

Apolipoprotein B

This gene encodes the structural protein of LDL particles responsible for the carriage of cholesterol through the blood. Mutations associated with APOB disrupt the binding of these LDL particles with LDLR, thus interfering with the clearance of cholesterol [8]. This disturbance can result in an increase in plasma LDL due to impaired clearance, a major risk factor for CAD. The most well-known mutation is APOB R3500Q, reducing the binding affinity of LDL particles to their receptors and present in autosomal dominant hypercholesterolemia [56].

Proprotein Convertase Subtilisin/Kexin Type 9 

The PCSK9 gene codes a specific protein involved in the regulation of LDL receptors. This protein binds with LDLR, resulting in its lysosomal degradation and reducing its availability on liver cell surfaces [10]. This mechanism increases the plasma level of LDL cholesterol and consequently increases the risk of developing CVD. Thus, while gain-of-function mutations of PCSK9 increase the degradation of LDL receptors and enhance hypercholesterolemia, mutations in PCSK9 that diminish its function lower the levels of LDL cholesterol and produce a reduced risk of CVD [57]. Because of the important role that PCSK9 plays in cholesterol metabolism, it has emerged as an exciting target for therapeutic intervention. PCSK9 inhibitors have been proven to be successful in lowering LDL cholesterol and lowering cardiovascular events [58].

Lipid metabolism regulation is critical to cardiovascular health and involves the pathways of LDLR, APOB, and PCSK9. Indeed, LDLR bears a critical role in the upkeep of cholesterol homeostasis. Upon binding of LDL cholesterol with LDLR on the surface of hepatocytes, it gets internalized-degraded and that way, cholesterol is utilized or else stored by the cell. The LDLR mutations that limit the function of LDLR result in poor clearance of LDL cholesterol through the bloodstream, leading to the promotion of lipid accumulation and the development of atherosclerosis [54]. This process is very important in understanding how genetic disorders like FH lead to the early development of CVD [55].

The role of PCSK9 in the regulation of LDLR availability has wide-ranging implications in lipid metabolism. PCSK9 binds to the LDLR and induces degradation in lysosomes, thereby preventing recycling of the receptor back to the cell surface. This reduces the number of available LDLRs for clearance of LDL cholesterol and results in consequentially increased levels of plasma LDL [10]. Gain-of-function mutations in PCSK9 have been associated with increased LDL cholesterol levels and increased risk for CVD, while loss-of-function mutations are characterized by decreased LDL levels and reduced risk of coronary events [57]. Currently, PCSK9 inhibitors interfering with a reciprocal interaction of PCSK9 with LDLR are extensively applied for enhancing LDL clearance and reduction of cardiovascular risk [58].

APOB is one of the important structural proteins in the constitution of the LDL particle, encoding a ligand that mediates the interaction of the LDL particles with LDLR [56]. Mutations here could make the LDL particles dysfunctional because they are much less efficient in binding with the LDL receptor, resulting in improper clearance of cholesterol and thus an increase in LDL levels. This pathway is particularly significant in those individuals who have mutations in both APOB and LDLR genes since their combined effects tend to aggravate the abnormalities in the lipids [8].

Inflammatory pathways, along with those related to lipid metabolism, are central to the initiation and progression of atherosclerosis. Cytokines such as interleukin- (IL-6) and tumor necrosis factor-alpha (TNF-α) promote endothelial dysfunction and plaque instability, precipitating clinical events like myocardial infarction [18]. Genetic predispositions in inflammatory mediators and genes controlling lipids elevate the risk of early-onset CVD by establishing a setting highly susceptible to both plaque development and rupture.

Genetic syndromes with cardiovascular components

Marfan Syndrome

Marfan syndrome is caused by a defect in connective tissue mainly caused by genetic mutations affecting the Fibrillin-1 (FBN-1) gene, encoding fibrillin-1, an indispensable constituent of the extracellular matrix. These mutations weaken the connective tissue, which in turn affects structures such as the cardiovascular system, especially the aorta [59]. Individuals with Marfan syndrome usually have aortic root dilation that may progress and lead to life-threatening aortic dissection if not treated [60]. Its cardiovascular implications are central to the diagnosis and management of this syndrome, wherein early recognition and surgical intervention significantly improve the outcome [61].

Ehlers-Danlos Syndrome

Ehlers-Danlos syndrome (EDS) is a genetically and clinically variable heterogeneous group of heritable disorders of connective tissue. In several subtypes, including the vascular Ehlers-Danlos syndrome (vEDS), mutations have been identified in the Collagen Type III Alpha 1 (COL3A1) gene, encoding for type III collagen, which is crucial for vascular and organ integrity [62]. vEDS is characterized by arterial fragility, with an increased risk of arterial rupture that usually presents as spontaneous rupture of an organ or vessel without previous aneurysm dilation [63]. These include arterial dissections and aneurysms, all of which require close monitoring and management of cardiovascular complications [59].

Advancements in genetic testing and screening

Genetic testing for CVD has experienced rapid progress involving various techniques for the identification of single genes and the whole genome. Single-gene tests search for mutations in one of several genes previously identified to cause inherited CVDs, such as LDLR, APOB, or PCSK9 genes in FH [64]. Normally, these tests are indicated in individuals when clinical findings strongly suggest a specific genetic condition [25].

Whole genome sequencing (WGS) and whole exome sequencing (WES) are more comprehensive approaches that analyze either part or the entire genome for genetic variations that could confer susceptibility to disease. WES focuses on the protein-coding regions of genes, estimated to harbor 85% of disease-causing mutations, and is, therefore, a cost-effective, targeted approach compared to WGS [65]. These new-generation sequencing technologies have facilitated the discovery of new genetic variants with access to complex gene interactions related to CVDs [66].

Thus, screening programs based on genetic testing have become prominent for the identification of individuals at high risk of CVD with early-onset features. Programs with multigene panels and next-generation sequencing technologies have generally been able to improve diagnosis and personalized medical management [9]. Including genetic screening in FH, clinics have improved their early diagnosis and initiation of preventive measures necessary for lipid-lowering therapy [26].

Genetic screening has also incorporated the polygenic risk assessment for common cardiovascular conditions into broader public health strategies. Such programs enhance stratification for prevention programs by identifying asymptomatic individuals with a high aggregate risk score [67]. The effectiveness of the screening programs often rests on the quality of the genetic counseling provided and the integration of genetic data with clinical risk factors [68].

The broadening of genetic testing to include early-onset CVD greatly increases ethical concerns. The major considerations concern the dilemma of informed consent, data privacy, and possible psychological consequences for the person receiving genetic risk information [22]. Challenges relevant to incidental findings reporting following WGS/WES concern pathogenic variants unanticipated and unrelated to CVD [69].

Other ethical issues include genetic discrimination regarding insurance or employment based on the individual profile of genetic risk, thus this issue emphasizes that the policies protecting against misuse of genetic information need to be developed. There are also questions concerning equity of access to advanced genetic testing that may widen health disparities if such services are accessible to only some populations [67].

Challenges and limitations in genetic studies for early-onset CVD

One of the major limitations in current genetic studies related to early-onset CVD has involved sample size considerations. Smaller sample sizes tend to be subject to limited statistical power, which restricts the capacity for the detection of subtle genetic associations [70]. Another significant challenge facing genetics is population diversity: most studies are disproportionately conducted on subjects of European descent [20]. This lack of representation impacts generalizability negatively and leaves non-European populations underrepresented, potentially worsening health disparities [71].

Genetic data interpretation is burdened with a significant risk of misunderstanding or misapplication. Variant of uncertain significance (VUS) mostly leads to indistinct clinical decisions that might pose difficulty for both the patient and the healthcare provider [72]. Overestimation of the implication of genetic findings, or misinterpretation of associations as causative, is associated with improperly informed health practices and futile psychological distress [73]. Genetic findings may also lead to deterministic views in which individuals consider that the presence of a particular genetic predisposition is a guarantee that disease will develop, without proper context provided for them [74].

Genetic testing for CVD may also have deep psychological consequences for a patient, particularly in cases of disclosure of high genetic risk. Genetic counseling can help explain accurate information to the patient and reassure anxiety or emotional reactions [75]. Genetic counselors are relatively scarce, and there is inconsistency in practices regarding the delivery of results, further contributing to confusion and emotional distress [76]. Besides, there are genetic discrimination concerns among patients in several spheres of life, such as at the workplace and insurance status, which affect the preference for testing [77].

Clinical implications and management strategies

Genetic information has rafted diametrically the strategy of early diagnosis and planning in the treatment of CVD. Identification of gene mutations and polygenic risk factors has enhanced the power of screening protocols. For example, WGS and WES have enabled the clinical identification of those individuals who are at higher risk due to genetic susceptibility mutations in LDLR, APOB, and PCSK9 [78]. Thus, the detection of this susceptibility can enable early treatment, such as lifestyle modification, and the possible early use of medication known to slow down the disease process [79].

Notably, even variable-onset disorders (e.g., COL3A1-related vEDS) demand early surveillance when genetic testing identifies high-risk youth, underscoring the need for age-adapted management protocols.

The advances in genetic testing have given rise to personalized medicine, where treatments can be designed according to an individual's genetic makeup. One of its subspecialties is pharmacogenomics, which predicts drug responses and potential adverse drug responses. This has been particularly useful in the management of lipid disorders and atherosclerotic conditions where genetic information guides the use of either statins or PCSK9 inhibitors [80]. Therapy that is tailored, based on one's genetic makeup, ensures efficacy in treatment and minimizes adverse events, thus improving patient outcomes [81].

Genetic discoveries have revolutionized the treatment for CVD due to targeted therapies. For instance, the development of PCSK9 inhibitors, such as alirocumab and evolocumab, was made possible by understanding the genetic mechanisms by which mutations of PCSK9 increase LDL cholesterol levels [82]. These inhibitors help lower LDL levels by 60% and are particularly useful in patients who do not show a suitable response to conventional statin therapy [83]. Moreover, gene therapy is a field that is still evolving, with studies into the use of CRISPR-Cas9 for editing faulty genes underlying cardiovascular risk, thus offering future potential for curative therapy [66].

While this review has focused on genetic disorders with well-characterized early-onset CVD manifestations (e.g., FH, HCM), we have also included conditions with broader age ranges (e.g., Marfan syndrome, LMNA cardiomyopathies) for two critical reasons. First, these disorders frequently precipitate life-threatening cardiovascular events in young adulthood (e.g., aortic dissection in Marfan syndrome), warranting their consideration in youth-focused risk stratification. Second, their molecular pathways (e.g., extracellular matrix defects in FBN-1) provide paradigmatic insights into mechanisms driving premature CVD, even in polygenic contexts. This approach aligns with growing recognition that age-of-onset classifications in genetic cardiology should incorporate both penetrance patterns and clinical urgency during high-risk life stages.

Future directions in genetic research for CVD

The new genetic technologies, above all CRISPR-Cas9, have the potential to transform the landscape in CVD research. It enables a precise modification in genes' DNA, which in turn can be utilized by researchers to repair pathogenic mutations that cause inherited CVD [84]. For example, the application of CRISPR has explored how effectively it can target and repair such genes as mutations of PCSK9, which in turn would reduce cholesterol levels along with the risk of atherosclerosis [85]. These, together with the continued development of more refined and safer gene-editing methodologies, are likely to point out new therapeutic strategies mauling the genetic components of CVD [86].

Multi-omics approaches have opened a new frontier in the elucidation of complex interplays of genomics, transcriptomics, proteomics, and metabolomics interaction in the pathogenesis of CVD [23]. By integrating these various levels of data, researchers could get overall insights into the disease mechanisms and identify new biomarkers for early diagnosis of diseases [19]. This approach depicts a more in-depth study of the interaction of genetic variations with environmental ones and gives a full view of the pathology of CVD [27].

Furthermore, DNA methylation and histone modification have been suggested as being among the major contributors to CVD [87]. Epigenetic changes may affect the level of expression of genes without actual changes in the genetic code; hence, states of epigenetics can have profound effects on disease outcomes [88]. Such advances might lead to targeted therapies that modulate epigenetic markers-opening up a whole new approach toward treatment and prevention [89].

CVD management requires an integrative approach of genetic insight with combinations of lifestyle and behavioral intervention [90]. Genetic predisposition can give way to personalized prevention plans, while changes in lifestyle, such as diet and exercise, might act to modulate gene expression in mitigating risk [22]. This synergy among genetic counseling, lifestyle adaptation, and targeted medical treatments has given way to a holistic strategy that maximizes the efficacy of managing cardiovascular risk [91]. Further research into the integration of these factors will doubtless result in an advance of precision medicine toward more flexible and personalized treatment options [92].

Table 1 presents a comprehensive summary of pathogenic mutations, their functional impacts, and clinical correlates in early-onset CVD.

**Table 1:** Summary of key genetic mutations and their effects LDLR: Low-Density Lipoprotein Receptor; LDL: Low-Density Lipoprotein; FH: Familial Hypercholesterolemia; APOB: Apolipoprotein B; CAD: Coronary artery disease; PCSK9: Proprotein Convertase Subtilisin/Kexin Type 9; FBN-1: Fibrillin-1; COL3A1: Collagen Type III Alpha 1; SCN5A: Sodium Voltage-Gated Channel Alpha Subunit 5; MYH7: Myosin Heavy Chain 7; HCM: Hypertrophic Cardiomyopathy; LMNA: Lamin A and C; TTN: Titin; TTNtv: Titin-Truncating Variants

Gene	Associated Mutation	Primary Effect	Clinical Implications	References
LDLR	Various loss-of-function mutations	Impaired functioning of the LDLR whereby there is poor clearance of LDL cholesterol from the blood	FH: risk for early-onset atherosclerosis	[7,54]
APOB	R3500Q and other mutations	Reduced binding of the LDL particles to the LDLRs, which in turn causes a decrease in the clearance of cholesterol	Autosomal dominant hypercholesterolemia and risk of CAD	[8,56]
PCSK9	Gain-of-function mutations	Increased degradation of LDLRs with consequent elevated levels of circulating LDL cholesterol	An increased risk of hypercholesterolemia and CAD	[10,57]
FBN-1	Missense and truncating mutations	Incorrect synthesis of FBN-1 results in weakened connective tissue	Aortic root dilation, risk of aortic dissection in Marfan syndrome	[59,61]
COL3A1	Mutations affecting type III collagen synthesis	Structural weaknesses in arterial and organ walls	Vascular Ehlers-Danlos syndrome, categorized by arterial ruptures	[62,63]
SCN5A	Missense mutations	Altered function of sodium channels in cardiac muscle	Increased risk for arrhythmias, including Brugada syndrome	[26,27]
MYH7	Missense mutations affecting myosin heavy chain	Contractility of cardiac muscle disrupted	HCM, risk of sudden cardiac death	[9,87]
LMNA	Various mutations affecting LMNA proteins	Abnormal nuclear envelope structure	Risk for arrhythmogenic cardiomyopathy and early-onset heart failure	[88,89]
TTN	Truncating mutations-TTNtv	Impaired function of the TTN protein, involved in sarcomere stability	Dilated cardiomyopathy, possible development of heart failure	[25,92]

## Conclusions

In conclusion, huge steps have been achieved in understanding the genetic underpinning for early-onset CVD, with the key knowledge derived from both monogenic and polygenic contributions. Key causative genes identified include LDLR, APOB, and PCSK9. Improvement in genetic testing, from single genes to comprehensive whole genome and exome sequencing, has enhanced early detection and provided specific treatment strategies. With personalized medicine, the integration of genetic findings into clinical practice has enabled detailed planning for treatment by targeted therapies, such as PCSK9 inhibitors. These clinical steps forward need to be balanced with considerations regarding ethical and psychological effects and disparities in access to genetic services. Genetics lessons reach deeply into the very relevance of public health itself. Effective screening programs that incorporate genetic risk factors could identify and prevent CVD much earlier than many of today's commonly used tools, which can help reduce the burden of disease and healthcare costs. This addition of genetic counseling and lifestyle interventions further supports holistic patient care, considering both genetic predispositions and modifiable risk factors. Further research and increased collaboration in genetic epidemiology will be warranted in the future. These include emerging technologies like CRISPR-Cas9 and multi-omics approaches, promising tools for future breakthroughs that will enable novel therapeutic strategies and extended risk assessment models. To accomplish this, it is necessary to expand diverse population studies so the benefits of genetic research are inclusive and equitably distributed. Better management in early-onset CVD, for this reason, requires an integrated approach that bridges genetics to clinical practice and public health.
